# Qualitative analysis and exploration of anti-inflammatory and antibacterial effects of a Thai traditional medicine formula from Wat Pho beyond its use for COVID-19 treatment

**DOI:** 10.1186/s12906-025-04873-3

**Published:** 2025-04-25

**Authors:** Naruemon Perstwong, Asma Binalee, Khwanlada Kobtrakul, Wongsakorn Phongsopitanun, Kittipong Sanookpan, Sudartip Areecheewakul, Visarut Buranasudja, Sornkanok Vimolmangkang

**Affiliations:** 1 Graduate Program in Pharmaceutical Science and Technology, Faculty of Pharmaceutical Sciences, Chulalongkorn University, Bangkok, 10330 Thailand; 2 Phyto Analytica Testing Laboratory, Leapdelab Company Limited, Bangkok, 10330 Thailand; 3 Department of Biochemistry and Microbiology, Faculty of Pharmaceutical Sciences, Chulalongkorn University, Bangkok, 10330 Thailand; 4 Nabsolute Company Limited, Bangkok, 10330 Thailand; 5 Department of Pharmacology and Physiology, Faculty of Pharmaceutical Sciences, Chulalongkorn University, Bangkok, 10330 Thailand; 6 Department of Pharmacognosy & Pharmaceutical Botany, Faculty of Pharmaceutical Sciences, Chulalongkorn University, Bangkok, 10330 Thailand; 7 Center of Excellence in Plant-Produced Pharmaceuticals, Chulalongkorn University, Bangkok, 10330 Thailand

## Abstract

**Background:**

Ya-Kao (YK) is a traditional Thai medicine used to relieve fever and consists of 14 crude drugs. It has been recommended by Thai folk healers and the Department of Thai Traditional and Alternative Medicine for COVID-19 treatment, with clinical studies conducted to assess its efficacy. However, scientific evidence on its biological properties remains limited. This study aims to explore the quality aspect of YK and evaluate its antibacterial and anti-inflammatory effects.

**Methods:**

The quality aspect of the YK formula was assessed using high-performance thin-layer chromatography (HPTLC). Agar disc diffusion and TLC bioautography were used for antibacterial activity, while anti-inflammatory effects were evaluated by measuring nitric oxide (NO) production in LPS-stimulated RAW264.7 macrophages.

**Results:**

The HPTLC method, utilizing a developing system of toluene, ethyl acetate, and formic acid (70:30:1, v/v/v), was successfully developed for the authentication of YK crude drugs. This method provides the most distinct fingerprint pattern of the components within the YK formulation, enabling clear visualization of its constituent. Additionally, it enables preliminary identification of phenolics, terpenes, and steroids, providing valuable insight into the formulation’s chemical composition. The antibacterial activity of YK was evaluated using the disk diffusion assay, and it was found that the lime juice extract of the YK formula (LYK) exhibited the largest zone of inhibition against both Gram-positive and Gram-negative bacterial strains, particularly those pathogenic to the respiratory tract. Additionally, the antibacterial activity was evaluated using TLC bioautography, and the results indicated that the methanolic extract of YK (MYK) exhibited an inhibition zone against *Streptococcus pyogenes* and *Klebsiella pneumoniae*. It was found that several compounds displayed an inhibition zone. Furthermore, the YK extract with methanol, water, and lime juice exhibited significant anti-inflammatory properties by suppressing NO accumulation in LPS-stimulated macrophage cells (*p* < 0.05).

**Conclusions:**

This study is the first to standardize YK raw materials using HPTLC and evaluate the biological properties of each crude drug and the combined formula. The developed HPTLC method ensures accurate identification of YK raw materials, preventing the use of incorrect ingredients. Additionally, the pharmacological findings confirm YK’s anti-inflammatory and antibacterial activities, particularly against respiratory pathogens linked to COVID-19.

**Supplementary Information:**

The online version contains supplementary material available at 10.1186/s12906-025-04873-3.

## Background

COVID-19 is an infectious disease caused by severe acute respiratory syndrome coronavirus 2 (SARS-COV-2). The common symptoms of COVID-19 include cough, difficulty breathing, sore throat, loss of taste, not feeling well, nausea, vomiting, and diarrhea. Additionally, many infected patients have been found to develop bacterial co-infections and secondary infections [1, 2]. During the COVID-19 pandemic, the healthcare system in Thailand faced significant challenges due to the increasing number of patients. The treatment protocols for confirmed COVID-19 cases are categorized into three levels based on disease severity: severe cases with pneumonia, mild cases in high-risk groups, and mild cases. COVID-19 treatment is based on modulating the immune response using anti-inflammatory drugs, and broad-spectrum antibiotic drugs, such as doxycycline or amoxicillin, were used for bacterial coinfection [3]. Patients with mild COVID-19 received supportive treatment without antiviral therapy and were quarantined at home. The Department of Thai Traditional and Alternative Medicine (DTAM), Ministry of Public Health (MOPH), recommended the use of traditional remedies, such as *Andrographis paniculate* (Burm) Wall. ex Ness., Ya-Ha-Rak (*Harrisonia perforate* (Blanco) Merr., *Clerodendrum indicum* (L.) Kubtze, *Capparis micracantha* DC., *Ficus racemose* L., and *Tiliacora triandra* (Colebr.) Diels.), and Ya-Kao (YK), as supportive treatments for mild cases of COVID-19 to reduce the number of hospitalized cases [4].

YK (in Thai, Ya means medicine and Kao means white color) is a Thai traditional medicine (TTM) for relieving fever, as recorded in an inscription at Wat Pho, a first-class royal temple, which was rebuilt by Phra Phutthayotfa Chulalok Maharaj (King Rama I). In TTM wisdom, herbal remedies for relieving fever were described in the Tak-Ka-Si-La Scripture. In TTM, fever is a symptom of high body temperature caused by infection. Fevers are categorized into several types based on signs, symptoms, and underlying causes, including those related to skin lesions, the nervous system, and seasonal changes [5]. According to the Wat Pho inscription, YK can treat bacterial infections, such as scrub typhus, typhoid fever, and scarlet fever, and viral infections, such as smallpox, chickenpox, herpes simplex, herpes zoster, sepsis, common cold, and influenza [4]. The YK formula is composed of the roots and stems of 14 plants: *Sauropus androgynus* (Linn.) Merr., *Breynia androgyna* (Phyllanthaceae), *Rhinacanthus nasutus* (L.) Kurz (Acanthaceae), *Merremia vitifolia* (Burm. f.) Hallier f. (Convolvulaceae), *Dregea volubilis* (L.f.) Benth. ex-Hook. (Apocynaceae), *Adenia viridiflora* Craib. (Passifloraceae), *Tiliacora triandra* (Menispermaceae), *Camellia sinensis* var. assamica (J.W. Mast.) Kitam. (Theaceae), *Citrus aurantifolia* (Christm.) Swing. (Rutaceae), *Combretum quadrangulare* Kurz. (Combretaceae), *Momordica cochinchinensis* (Lour.) Spreng. (Cucurbitaceae), *Glochidion zeylanicum* (Gaertn.) A. Juss (Phyllanthaceae), *Schumannianthus dichotomus* (Roxb.) Gagnep./*Schumannianthus benthamianus* (Marantaceae), *Hydnophytum formicarum* Jack (Rubiaceae), and *Caesalpinia bonduc* (L.) Roxb. (Caesalpiniaceae). In traditional wisdom, the dried powder of YK was suspended in recommended liquid vehicles, either rice-washing water as an antipyretic agent or lime juice as an anticough and expectorant agent [4, 5]. In the TTM pharmacy textbook, 32 types of liquid vehicles were documented. Rice-washing water was used to alleviate dizziness and nausea, whereas lime juice was used to treat dried sputum [6].

The acceptance of YK in clinical practice was based on its proven effectiveness, as recorded by a traditional Thai doctor who used YK to treat patients with COVID-19 at the Phoreang TTM Clinic in Samut Sakhon Province. The doctor conducted a study on patients from three groups: those with mild cases, those with pneumonia, and healthy individuals (to prevent infection). All patients took 1 teaspoon of YK powder suspended in ½ lime juice and warm water three times a day after meals for 14 consecutive days and were followed up every 3 days. The results showed that the infection was treated after 6 days. Therefore, this study revealed the ability of YK to prevent and treat COVID-19 infection [7]. However, the biological activity of the YK formula has not been reported. Of the 14 plants in the formula, some plants have been reported to have biological properties. For example, the root of *T. triandra* has antioxidant, anti-inflammatory [8], antiplasmodial [9, 10], anti-lung cancer [11], antipyretic, and antimicrobial properties [12]. The dried leaf extract of *S. androgynus* has antioxidant, antidiabetic, and antihemolytic properties [13]. The leaf extract of *C. sinensis* has antioxidant, antidiabetic, and anti-inflammatory properties [14], whereas the ethanolic extract of *C. aurantifolia* has anti-liver cancer properties [15]. Therefore, the YK formula has antibacterial and anti-inflammatory effects against secondary infections.

Authenticating raw materials to ensure the quality of traditional medicines composed of several plants is an important issue. It is necessary to prevent the wrong selection of ingredients during product preparation and to screen the biological effects of the YK formula. Therefore, this study aimed to identify the chemical profiles of crude drugs from the YK formula and YK product using high-performance thin-layer chromatography (HPTLC) and investigate the antibacterial and anti-inflammatory properties of the formula and its crude drugs to provide supporting evidence for the efficacy of the YK remedy.

## Methods

### Chemical reagents

For HPTLC analysis, the following reagents and solvents were obtained: ethanol (Cat no. 64175), methanol (Cat no. 34860), and toluene (Cat no. 108883) from Millipore Sigma® in Germany, formic acid (98%–100%, Cas no. 64186), acetic acid (Code No. 1.00063.2500), ethyl acetate (Code No. 1.09623.2500), and hexane (Code No. 1.04367.2500) from Merck®, diethyl amine (Code No. 8.03010.0500) from Sigma Aldrich®, chloroform AR (21 09 0024) and 1-butanol (AR1024) from RCI Labscan® in Bangkok, Thailand, and sulfuric acid (Code No. 1.00731.2500) and phosphoric acid (Code No. 1.00573.1000) from Sapelo® in Germany. Derivative reagents, such as anisaldehyde, ninhydrin, sulfuric acid, and Dragendorff TS2, were prepared (Table 2). For the anti-inflammatory assessment, Griess reaction components, including sulfanilamide (Cas RN®. 63-74-1), N-1-naphthylethylenediamine dihydrochloride (NED) (Cas RN®. 1465-25-4), and sodium nitrite (Cas RN®. 7632-00-0), were purchased from Tokyo Chemical Industry Co., Ltd., Japan. The crude extract was dissolved in dimethyl sulfoxide (DMSO) purchased from Carlo Erba Reagents GmbH, Dasit Group, Italy (Batch No. P1L450181L).

### Plant materials

**Fig. 1:**
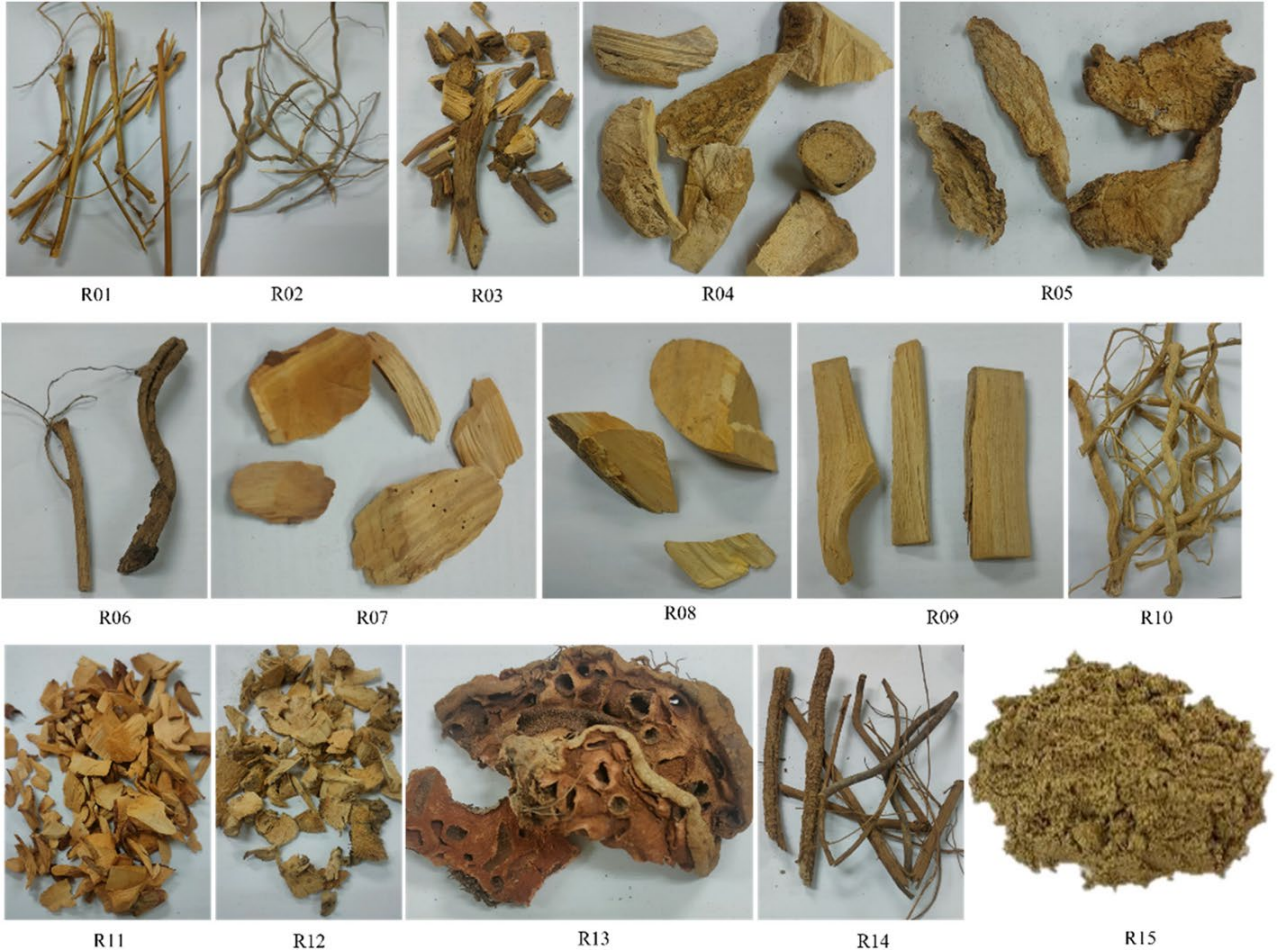
Crude drugs of the YK formula and the YK product. R01, the root of *S. androgynus*; R02, the root of *R. nasutus* (L.); R03, the root of *M. vitifolia*; R04, the root of *D. volubilis*; R05, the root of *A. viridiflora*; R06, the root of *T. triandra*; R07, the root of *C. sinensis*; R08, the root of *C. aurantifolia*; R09, the root of *C. quadrangulare*; R10, the root of *M. cochinchinensis*; R11, the root of *G. zeylanicum*; R12, the root of *S. dichotomus*; R13, the stem of *H. formicarum*; R14, the root of *C. bonduc*; and R15, the YK product

The YK formula comprises 14 crude drugs (Table 1). Crude drugs (R01–R14) and the YK product (No. R15) (Fig. 1) were procured from Innovative Pharma Herbs Company Limited, Phetchaburi Province, Thailand. The authenticity of the samples was confirmed by the DTAM, MOPH, Nonthaburi, Thailand. Voucher specimens for each crude drug were deposited at DTAM, MOPH, Thailand.

**Table 1:** Crude drugs in the YK product

No	Scientific name (family)	Synonym	Thai name	Part use	Voucher no
R01	*Sauropus androgynus* (Linn.) Merr. (Phyllanthaceae)	*Breynia androgyna*	Phak wan ban	Root	TTM No. 1000793
R02	*Rhinacanthus nasutus* (L.) Kurz (Acanthaceae)	-	Thong phan chang (white crane flower)	Root	TTM No. 1000794
R03	*Merremia vitifolia* (Burm. f.) Hallier f. (Convolvulaceae)	-	Chingcho lueang (morning glory)	Root	TTM No. 1000795
R04	*Dregea volubilis* (L.f.) Benth. ex-Hook. (Apocynaceae)	-	Kra thong ma ba or huan mu	Root	TTM No. 1000796
R05	*Adenia viridiflora* Craib. (Passifloraceae)	-	Phak sap	Root	TTM No. 1000797
R06	*Tiliacora triandra* (Menispermaceae)	-	Ya-nang	Root	TTM No. 1000798
R07	*Camellia sinensis* var. assamica (J.W. Mast.) Kitam. (Theaceae)	-	Cha, miang, or tea plant	Root	TTM No. 1000799
R08	*Citrus* × *aurantifolia* (Christm.) Swingle (Rutaceae)	-	Ma nao or lime	Root	TTM No. 1000800
R09	*Combretum quadrangulare* Kurz. (Combretaceae)	-	Sakae na	Root	TTM No. 1000801
R10	*Momordica cochinchinensis* (Lour.) Spreng. (Cucurbitaceae)	-	Fak khao, baby jackfruit, or gac	Root	TTM No. 1000802
R11	*Glochidion zeylanicum* (Gaertn.) A. Juss (Phyllanthaceae)	-	Umbrella cheese tree, chumset, som set, or khrai mot	Root	TTM No. 1000803
R12	*Schumannianthus dichotomus* (Roxb.) Gagnep (Marantaceae)	*S. benthamianus*	Khla	Root	TTM No. 1000804
R13	*Hydnophytum formicarum* Jack (Rubiaceae)	-	Ant plant, hua roi ru, or krachao phimot	Stem	TTM No. 1000805
R14	*Caesalpinia bonduc* (L.) Roxb (Caesalpiniaceae)	-	Nicker bean or sawat	Root	TTM No. 1000806

### Sample preparation of the YK formula

#### Crude drug extracts

10 g of the dried powder of each crude drug (R01–R14) was subjected to extraction using 100 mL of methanol. The extraction process involved sonication for 15 min at room temperature and centrifugation at 5,000 rpm for 5 min to eliminate the powder residue. The resulting supernatant was evaporated to dryness using a rotary evaporator. The chemical profiles of the dried extracts were analyzed using HPTLC, and their biological activities were evaluated.

#### YK extracts

20 g of dried powder of the YK product was subjected to extraction using 200 mL of various liquid vehicles (water, lime juice, rice-washing water, and methanol) following the Thai Traditional Knowledge and conventional solvent methods. The extraction process involved sonication for 15 min at room temperature and centrifugation at 5,000 rpm for 5 min. The resulting extracts were processed as follows: water extract of YK (WYK), lime juice extract of YK (LYK), and rice-washing water extract of YK (RYK) were lyophilized to dryness, whereas the methanol extract of YK (MYK) was evaporated using a rotary evaporator. The antibacterial and anti-inflammatory activities of these extracts were evaluated.

### HPTLC analysis

Each of the crude extracts, totaling 20 mg, was dissolved in 1 mL of methanol within 1.5 mL microcentrifuge tubes (Lot no. 6034911; Quality Scientific Plastics, China). 4 µL/band of each crude extract was applied to HPTLC glass plates (20 × 10 cm, Merck silica gel 60 F254) using a semiautomatic applicator (Linomat 5, Camag, Muttenz, Switzerland). Each band was 8 mm wide, positioned 11.4 mm apart, and situated 8 mm from the lower edge and 20 mm from the left edge of the plate. The plates were developed within a chamber saturated with mobile phase vapor. Four mobile phase systems (Table 2) were used for the YK formula, whereas chloroform and ethanol (70:30) were used for Ya-nang [16]. Development proceeded with a migration distance of 70 mm using an automatic development chamber ADC2 (Camag; Muttenz, Switzerland). The plate was heated to 100 °C for 2 min using a TLC Plate Heater (Camag; Muttenz, Switzerland) and sprayed with a specific reagent to observe the chemical profile of the crude extract (Table 2). The derivatized plates were visualized under white light, ultraviolet (UV) light at 254 nm, and UV light at 366 nm using Visualizer 2 (Camag; Muttenz, Switzerland).

**Table 2:** Summary of developing solvents in the HPTLC techniques used in this experiment [17–19]

Developing system code	Developing solvents	Ratio (v/v/v)	Derivatization reagents	Detection
A	Toluene, ethyl acetate, and formic acid	70:30:1	Anisaldehyde (1 mL of *p*-anisaldehyde in 10 mL of sulfuric acid, 20 mL of acetic acid, and 170 mL of ice-cool methanol)	Phenolic, sugar, steroids, terpenes, and apolar flavonoids
B	1-Butanol, acetic acid, and water	7:1:2	Ninhydrin (0.2% v/v ninhydrin (2,2-dihydroxyindene- 1,3-dione) in 96% ethanol and 3 mL of glacial acetic acid)	Amino acid, amines, aminoglycoside antibiotics, and peptide
C	Toluene, ethyl acetate, and diethyl amine	7:2:1	Dragendorff TS (solution A: 0.85 g of basic bismuth nitrate in 10 mL of glacial acetic acid and 40 mL of water; solution B: 8 g of potassium iodide in 30 mL of water)	Alkaloids
D	Hexane and ethyl acetate	2:1	Sulfuric acid reagent (10% v/v sulfuric acid (98%) in methanol under cooling)	Naphthoquinone and lignin

### Microorganisms and culture media

In this experiment, both Gram-positive and Gram-negative bacterial strains were used as the tested microorganisms. The Gram-positive bacteria included *Kocuria rhizophila* American Type Culture Collection (ATCC) 9341*, Staphylococcus aureus* ATCC 25923, *S. aureus* DMST 20646, *Bacillus subtilis* ATCC 6633, methicillin-resistant *Staphylococcus aureus* (MRSA) DMST 20646, *Staphylococcus epidermidis* ATCC 12228, *Streptococcus pyogenes* DMST 4369, and *Streptococcus sobrinus.* The Gram-negative bacteria included *Escherichia coli* ATCC 25922, *Shigella* sp., *Enterococcus aerogenes*, *Klebsiella pneumoniae* ATCC 13883, and *Pseudomonas aeruginosa* ATCC 9027. All bacterial strains were cultured and maintained on Mueller–Hinton agar (MHA) at 37 °C for 24 h. Before the antimicrobial assay, a single colony of cultured bacteria was transferred into a 0.85% (w/v) normal saline solution to achieve a turbidity concentration within the range of the 0.5 McFarland standardized solution.

### Antimicrobial activity assay using disk diffusion agar

The antimicrobial activity was evaluated using the disk diffusion agar method, following standard guidelines and previously published reports [20, 21]. 20 µL of the samples (WYK, LYK, RYK, and MYK) was loaded onto 6 mm sterilized paper disks. The concentrations of these samples were 1,000 mg/mL for the YK formula extracts and 100 mg/mL for the raw plant crude extracts. DMSO was used as the negative control, and 30 µg/disk of gentamicin served as the positive control. The paper disks loaded with the samples were placed onto MHA swabbed with a bacterial solution with a turbidity concentration of 0.5 McFarland. The cultures were incubated at 37 °C for 24 h, and the inhibition zones were recorded.

### Thin-layer chromatography (TLC) bioautography for antimicrobial activity

The antimicrobial activity was evaluated using the TLC bioautography method, adapted from previously published reports [22]. *S. pyogenes* DMST 4369 and *K. pneumoniae* ATCC 13883 were chosen as the target bacteria. 10 µL of the MYK formula and crude drug extracts (at a concentration of 50 mg/mL) were applied to aluminum TLC silica gel 60 F254 plates measuring 20 × 10 cm. The same conditions as those used for HPTLC were applied, employing solvent systems A and D. The TLC plate was briefly dipped into a bacterial suspension in Mueller–Hinton broth (MHB) with a turbidity concentration of 0.5 McFarland for 10 s. Subsequently, the TLC plate containing the samples was placed onto MHA. The TLC plate was incubated at 37 °C for 24 h. After incubation, the TLC plate was sprayed with a 0.2% v/v solution of iodonitrotetrazolium chloride (INT) dye to facilitate the detection of bacterial growth. The inhibition zone, which appeared around the red background (indicating the growth area), was observed.

### Cell culture

RAW264.7 mouse macrophages were purchased from the American Type Culture Collection (ATCC). Cells were cultured in Dulbecco’s Modified Eagle Medium supplemented with 10% v/v fetal bovine serum, 10% U/mL penicillin, and 100 µg/mL streptomycin. The cell cultures were maintained at 37 °C under 5% CO_2_ condition. All cell culture media and supplements were obtained from Gibco™, Thermo Fisher Scientific Inc.

### 3-(4,5-Dimethylthiazol-2-yl)-2,5-diphenyltetrazolium bromide (MTT) assay

Cell viability was determined using the MTT assay, a method adapted from a previously published report [23, 24]. RAW264.7 cells were seeded in a 96-well plate at a density of 10,000 cells/well and allowed to culture for 24 h before treatment initiation. Following the specified treatments, the experimental medium was removed, and cells were rinsed twice with phosphate-buffered saline. RAW264.7 cells were treated with various sample concentrations, including 50–1,000 µg/mL of MYK, RYK, LYK, and WYK and 50 µg/mL of the crude extract overnight in a control incubator at 37 °C under 5% CO_2_. In contrast, untreated cells were used as the control group. Rinsed cells were incubated with 100 µl of MTT solution (0.4 mg/mL) in the dark in an incubator for 2 h. The MTT solution was gently aspirated, and the formazan crystals were solubilized in DMSO. The absorbance of the formazan solution was measured at a wavelength of 570 nm using a CLARIO Star microplate reader.

### Measurement of nitric oxide (NO) production

The measurement of anti-inflammatory activity of YK was performed using a method adapted from a previously published report [25–27]. RAW246.7 cells were seeded in a 24-well plate at a density of 25,000 cells/well and incubated for 24 h. The cells were treated with the sample under a control condition similar to that of the MTT assay. The concentrations of MYK, RYK, LYK, and WYK were 50–250 µg/mL, and those of the crude extracts were 50 µg/mL. Untreated cells were used as the control group. After overnight incubation, the experimental medium was harvested and used to measure the NO level. Following the indicated treatments, 100 µl of the experimental media was combined with 100 µl of Griess reagents (50 µl of 1% (w/v) sulfanilamide in 5% (v/v) phosphoric acid and 50 µl of 0.1% (w/v) NED). After 10 min of reactions, the colored azo compound byproduct resulting from the Griess reaction was quantified using a CLARIO Star microplate reader at UV absorbance at 540 nm.

### Statistical analysis

Data were presented as mean ± standard deviation (SD) from three replications and were analyzed using GraphPad Prism. The statistical differences between the control and treatment groups were assessed using one-way ANOVA. A *p*-value < 0.05 indicated statistical significance.

## Results

### Chromatographic analysis of the crude drug extracts from the YK formula by HPTLC

Herbal medicines lack control over the quality of raw materials, herbal drug preparations, and finished products [19]. The YK formula is a personalized medicine prepared by a TTM doctor, and the DTAM stated that it could prevent and treat COVID-19 infection. Therefore, YK did not have a standard quality control profile for the raw materials and the product. All crude drugs must be identified to ensure that the correct species are identified before drug preparation to identify the chemical fingerprint of the YK formula.

**Fig. 2:**
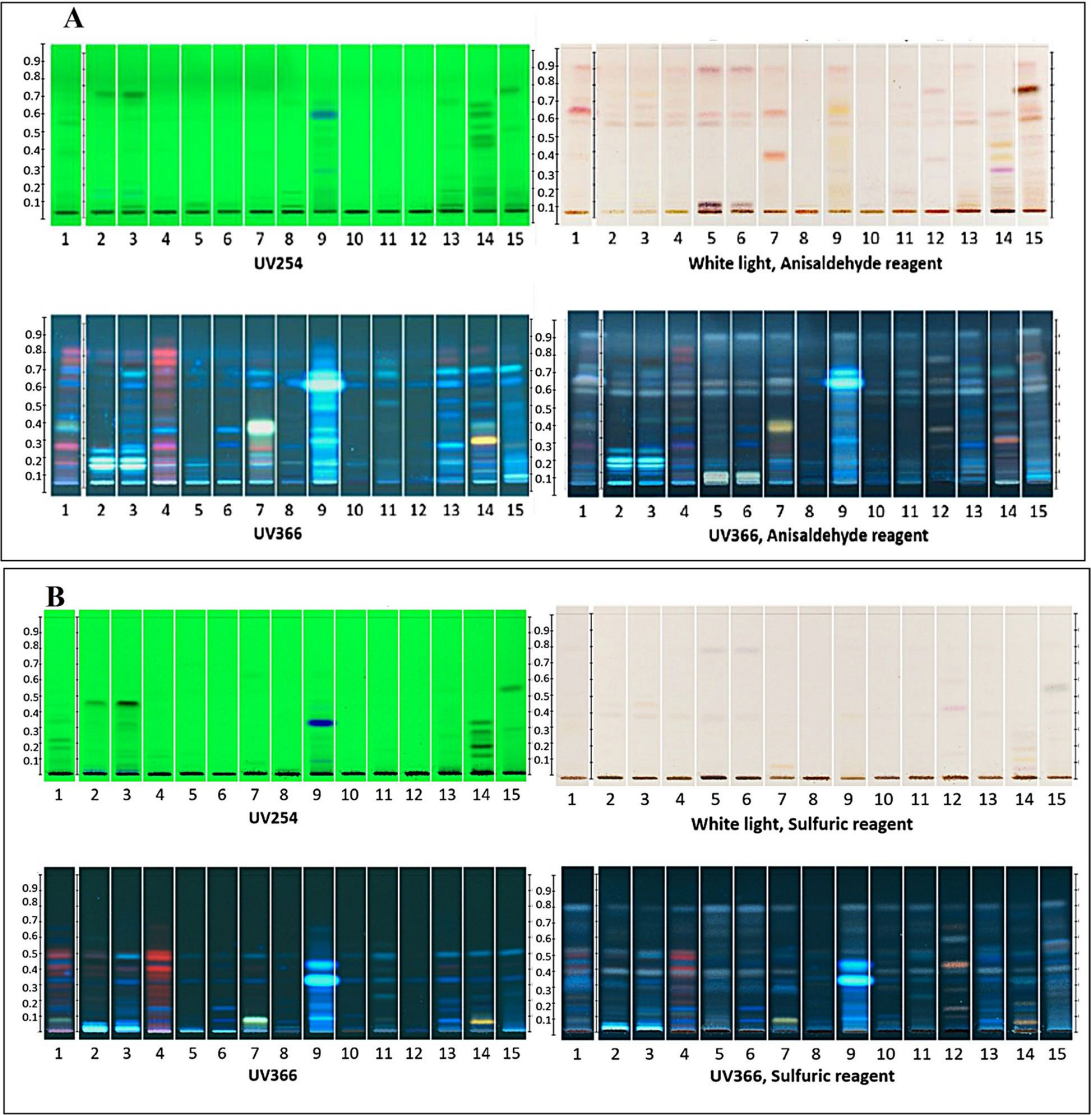
Chemical profiles of the YK formula and their ingredients. The mobile phase systems are toluene, ethyl acetate, and formic acid (70:30:1) (**A**) and hexane and ethyl acetate (2:1) (**B**). Track No. 1, the YK formula; Track No. 2, the root of *S. androgynous* and *B. androgyna;* Track No. 3, the root of *R. nasutus*; Track No. 4, the root of *M. vitifolia*; Track No. 5, the root of *D. volubilis*; Track No. 6, the root of *A. viridiflora*; Track No. 7, the root of *T. triandra*; Track No. 8, the root of *C. sinensis*; Track No. 9, the root of *C. aurantifolia*; Track No. 10, the root of *C. quadrangulare*; Track No. 11, the root of *M. cochinchinensis*; Track No. 12, the root of *G. zeylanicum*; Track No. 13, the root of *S. dichotomus/S. benthamianus*; Track No. 14*,* the stem of *H. formicarum*; and Track No. 15, the root of *C. bonduc*

Four mobile phases were used in this experiment to analyze the YK formula and its ingredients (Table 2). Two mobile phase systems were used for the chemical fingerprinting of the raw materials, which revealed a pattern of chemical profiles related to the YK formula. The results are shown in Fig. 2. One developing mobile phase was toluene, ethyl acetate, and formic acid (70:30:1), which is a standard method for detecting apolar flavonoid compounds [28]. Observation of the HPTLC bands of YK and all crude drug extracts under white light with anisaldehyde reagent and UV 366 nm with anisaldehyde reagent showed the separation of gray or green bands at R_f_ 0.5 and blue bands at R_f_ 0.6, except for the root of *C. sinensis* (Track No. 8), where the band was pale color (Fig. 2A). The appearance of gray or green bands and blue coloration after derivatization with anisaldehyde reagent may indicate the presence of phenolic, terpene, or steroid compounds [29].

Another developing mobile phase is hexane and ethyl acetate (2:1), which is selected to detect naphthoquinone and lignan compounds in this study. It is because this mobile phase was used as a standard method to detect phyllanthin and hypophyllanthin, which are major lignan compounds found in *Phyllanthus amarus* [17]. Observation of the HPTLC band of YK and all crude extracts under white light with sulfuric reagent and UV 366 nm with sulfuric reagent showed the separation of gray-brownish bands at R_f_ 0.48 and 0.61, except for the root of *C. sinensis* (Track No. 8), where the band was pale color (Fig. 2B).

**Fig. 3:**
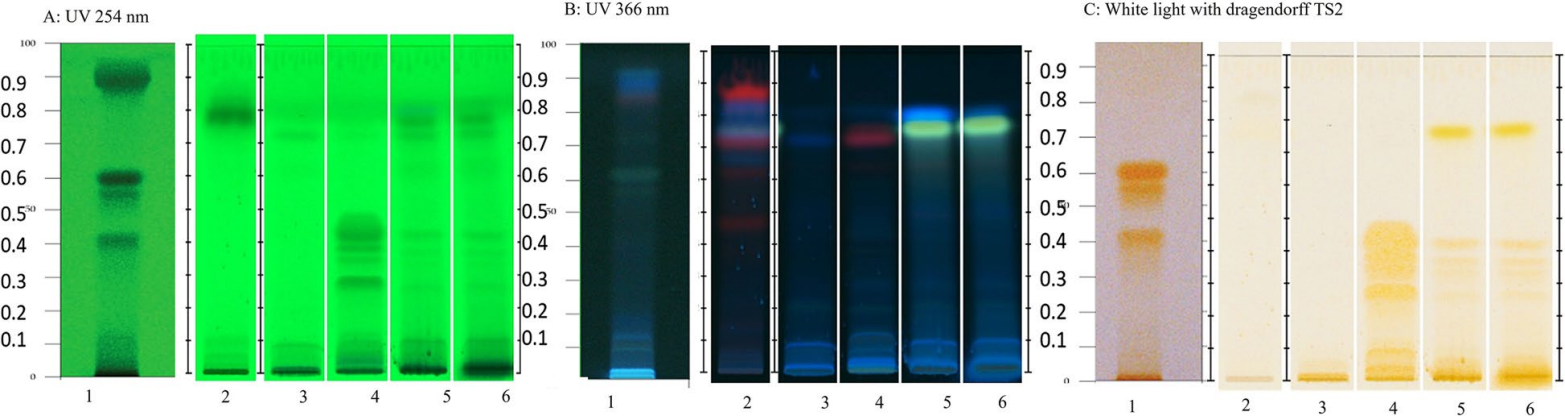
Chemical profile of *Tiliacora triandra* Diels (Menispermaceae), the main crude drug in the YK formula, by HPTLC under UV light at 254 nm and 366 nm and white light after derivatization with Dragendorff TS2. Chloroform:ethanol (70:30) was the solvent development system following THP 2021. Track No. 1, referenced data from THP 2021 result; Track No. 2, YK formula; Track No. 3, Ya-nang from the dispensary; Track No. 4, *T. triandra* root, which is a crude drug collected from a herbal garden in the Faculty of Pharmaceutical Sciences, Chulalongkorn University; and Tracks Nos. 5–6, *T. triandra* root, which is a crude drug provided by DTAM

One of the problems observed in this study was the high possibility of misidentifying Ya-nang, which is in the YK formula and is widely used in other antipyretic drugs in TTM. Ya-nang plants have two species based on TTM knowledge: *T. triandra* (Menispermaceae), Ya-nang kao, or Ya-nang and *Bauhinia strychnifolia* Craib (Fabaceae) or Ya-nang dang. Therefore, two authentic *T. triandra* samples obtained from DTAM and the Faculty of Pharmaceutical Sciences, Chulalongkorn University, were compared with one Ya-nang sample purchased from a drug dispensary store and the YK formula to identify the species of Ya-nang in the YK formula. Following the Ya-nang monograph in the Thai Herbal Pharmacopoeia (THP), the mobile phase of TLC is chloroform and ethanol (70:30, % v/v). Figure 3 shows the chemical profiles. The data from the Ya-nang monograph in THP (Track No. 1) showed three orange bands (R_f_ 0.40, 0.55, and 0.60) after derivatization with Dragendorff TS2, which may be bisbenzylisoquinoline alkaloids, which are the main constituent compounds in *T. triandra* root [16]. Furthermore, it should be noted that the HPTLC plate was used in this study, whereas the THP monograph used the TLC plate. Therefore, the overall bands in this study (Tracks Nos. 2–6) showed lower R_f_ than the reference data (Track No. 1). The authentic *T. triandra* from both sources (Tracks Nos. 4–6) exhibited a similar pattern of orange bands after derivatization as that of the reference. An additional yellow band at R_f_ 0.78 under UV366 light was observed only in *T. triandra* from DTAM (Tracks Nos. 5–6). Unexpectedly, Ya-nang from the dispensary (Track No. 3) did not match the HPTLC profile of the reference Ya-nang. Although the YK formula (Track No. 2) did not show a chemical fingerprint after derivatizing with Dragendorff TS2, the bands at R_f_ 0.78 under UV366 nm and UV 254 nm were similar to the authentic *T. triandra* samples. This may be because the YK formula includes 14 ingredients, leading to the low intensity of the bands.

### Antimicrobial activity by agar disk diffusion test

A high concentration (100 mg/ml) was used to confirm the antibacterial activity of the YK extract and crude drug extracts. This confirms that antimicrobial activity would be detectable if the YK extract and crude drug extracts exhibited antimicrobial activity. If no inhibition zone was observed even at higher concentrations, this suggests that the extracts likely do not possess antimicrobial activity against the tested microorganisms.

The 14 crude drug extracts of the YK formula at a concentration of 100 mg/mL in methanol were screened for the antibacterial activity. Table 3 and Supplementary Figures S1–3 show the results of the antibacterial activity of each crude drug. The methanolic extract of 10 crude drugs in the YK formula exhibited antibacterial activity. Specifically, the methanolic extracts of *C. quadrangulare*, *M. cochinchinensis*, *G. zeylanicum*, and *H. formicarum* inhibited 4 bacterial strains: *S. aureus*, MRSA, *S. epidermidis*, and *S. sobrinus.* Additionally, *R. nasutus*, *C. quadrangulare, M. cochinchinensis*, *G. zeylanicum*, and *H. formicarum* demonstrated antibacterial activity against *S. epidermidis* and *S. pyogenes*. *S. androgynus, B. androgyna,* and *M. vitifolia* exhibited antibacterial activity against *S. pyogenes* only, whereas *T. triandra*, *C. sinensis,* and *S. dichotomus/S. benthamianus* exhibited activity against *S. epidermidis* only. Moreover, lime juice exhibited antibacterial activity against both Gram-positive and Gram-negative bacteria strains, including *S. aureus,* MRSA*, S. epidermidis, S. pyogenes, S. sobrinus, E. coli, K. pneumoniae, P. aeruginosa,* and *E. aerogenes*. A comparison of the antibacterial activities of lime juice and LYK extract revealed that LYK extract had a higher inhibition zone than lime juice. This indicates that lime juice should be used as a liquid vehicle to suspend the YK formula and achieve a synergistic effect.

**Table 3:** Antibacterial activity of the YK extract in different Traditional Thai liquid vehicles and methanol by disk diffusion test

Bacteria strains	Inhibition zone (mm) of samples (mean ± SD)				
	MYK	LYK	RYK	WYK	Gentamycin 30 µg
**Gram-positive bacteria**					
*S. aureus* ATCC 25923	9.50 ± 0.00	16.73 ± 0.32	-	-	34.50
*S. aureus* 6538	7.30 ± 0.00	11.67 ± 0.29	-	-	26.00
MRSA DMST 20646	8.03 ± 0.12	21.83 ± 0.76	-	-	-
*B. subtilis* ATCC 6633	8.03 ± 0.06	14.90 ± 0.00	-	-	28.90
*S. epidermidis* ATCC 12228	-	14.83 ± 0.29	-	-	33.00
*S. pyogenes* DMST 4369	7.93 ± 0.40	17.73 ± 0.21	-	-	25.00
*S. sobrinus*	-	22.67 ± 0.29	-	-	38.50
*K. rhizophila* ATCC 9341	9.10 ± 0.36	24.00 ± 1.32	-	-	34.50
**Gram-negative bacteria**					
*Shigella* sp.	-	9.43 ± 0.40	-	-	26.50
*E. coli* ATCC 25922	-	10.00 ± 0.00	-	-	25.00
*K. pneumoniae* ATCC 13883	-	17.67 ± 0.29	-	-	32.00
*P. aeruginosa* ATCC 9027	-	11.77 ± 0.40	-	-	26.30
*E. aerogenes*	-	11.07 ± 0.06	-	-	25.70

-, no zone of inhibition

In preliminary experiments, 100 mg/ml of MYK, LYK, RYK, and WYK was investigated for their antibacterial activity by agar disk diffusion. However, no inhibition zone was observed. The extracts were increased to 1,000 mg/mL to detect any potential antimicrobial activity, which resulted in the appearance of an inhibition zone, indicating antimicrobial effects. The results showed the activity of 1,000 mg/ml MYK, LYK, RYK, and WYK against eight Gram-positive and five Gram-negative bacteria. After incubation overnight at a control temperature of 37 °C, the results showed that LYK exhibited an inhibition zone on all strains, whereas MYK exhibited an inhibition zone on only 7 Gram-positive strains. However, RYK and WYK did not show the effect on all strains. Table 3 shows that LYK had stronger antibacterial activity than MYK, RYK, and WYK. Furthermore, LYK was chosen to assess its antibacterial activity in comparison with the ingredients in the YK formula to identify the effects. LYK exhibited the highest inhibition zone on *K. rhizophila* ATCC 9341*, S. sobrinus,* and MRSA DMST 20646 at 24.00 ± 1.32, 22.67 ± 0.29, and 21.83 ± 0.76, respectively (Table 4). Moreover, it showed inhibitory activity against respiratory tract infection-related bacteria, including *S. pyogenes* DMST 4369, *S. aureus* ATCC 25923, *S. aureus* 6538, *K. pneumoniae* ATCC 13883, and *P. aeruginosa* ATCC 9027.

**Table 4:** The inhibition zone of LYK, methanolic extract of the crude drug, and lime juice at a concentration of 100 mg/mL

Strains	**Inhibition zone (mm) of samples**																
	No. 1	No. 2	No. 3	No. 4	No. 5	No. 6	No. 7	No. 8	No. 9	No. 10	No. 11	No. 12	No. 13	No. 14	No. 15	LYK	Gentamicin 30 µg
*S. aureus* ATCC 25923	-	-	-	-	-	-	-	-	12.25	8.65	7.55	-	8.10	-	12.00	16.73	27.75
*S. aureus* 6538	-	-	-	-	-	-	-	-	18.00	8.55	-	-	-	-	8.30	11.67	24.1
MRSA DMST 20646	-	-	-	-	-	-	-	-	15.35	8.45	11.10	-	10.30	-	9.15	21.83	-
*S. epidermidis* ATCC 12228	-	8.10	-	-	-	10.60	12.25	-	20.10	12.70	15.05	8.00	10.25	-	12.00	14.83	31.30
*S. pyogenes* DMST 4369	7.30	8.30	9.75	-	-	-	-	-	-	-	-	-	-	-	20.0	17.73	30.25
*S. sobrinus*	-	-	-	-	-	-	-	-	18.25	7.10	8.55	-	9.00	-	12.08	22.67	33.50
*Shigella* sp.	-	-	-	-	-	-	-	-	-	-	-	-	-	-	-	9.43	28.00
*Salmonella* sp.	-	-	-	-	-	-	-	-	-	-	-	-	-	-	-	n/a	21.75
*E. coli* ATCC 25922	-	-	-	-	-	-	-	-	-	-	-	-	-	-	9.75	10	24.25
*K. pneumoniae* ATCC 13883	-	-	-	-	-	-	-	-	-	-	-	-	-	-	9.75	17.67	329
*P. aeruginosa* ATCC 9027	-	-	-	-	-	-	-	-	-	-	-	-	-	-	9.25	11.77	25.7
*E. aerogenes*	-	-	-	-	-	-	-	-	-	-	-	-	-	-	8.25	11.07	25.25

No. 1, *S. androgynus;* No. 2, *R. nasutus;* No. 3, *M. vitifolia*; No. 4, *D. volubilis*; No. 5, *A. viridiflora*; No. 6, *T. triandra*; No. 7, *C. sinensis*; No. 8, *C. aurantifolia*; No. 9, *C. quadrangulare*; No. 10, *M. cochinchinensis*; No. 11, *G. zeylanicum*; No. 12, *S. dichotomus;* No. 13, *H. formicarum*; No. 14, *C. Bonduc;* and No. 15, lime juice

### Antimicrobial activity by TLC bioautography

**Fig. 4:**
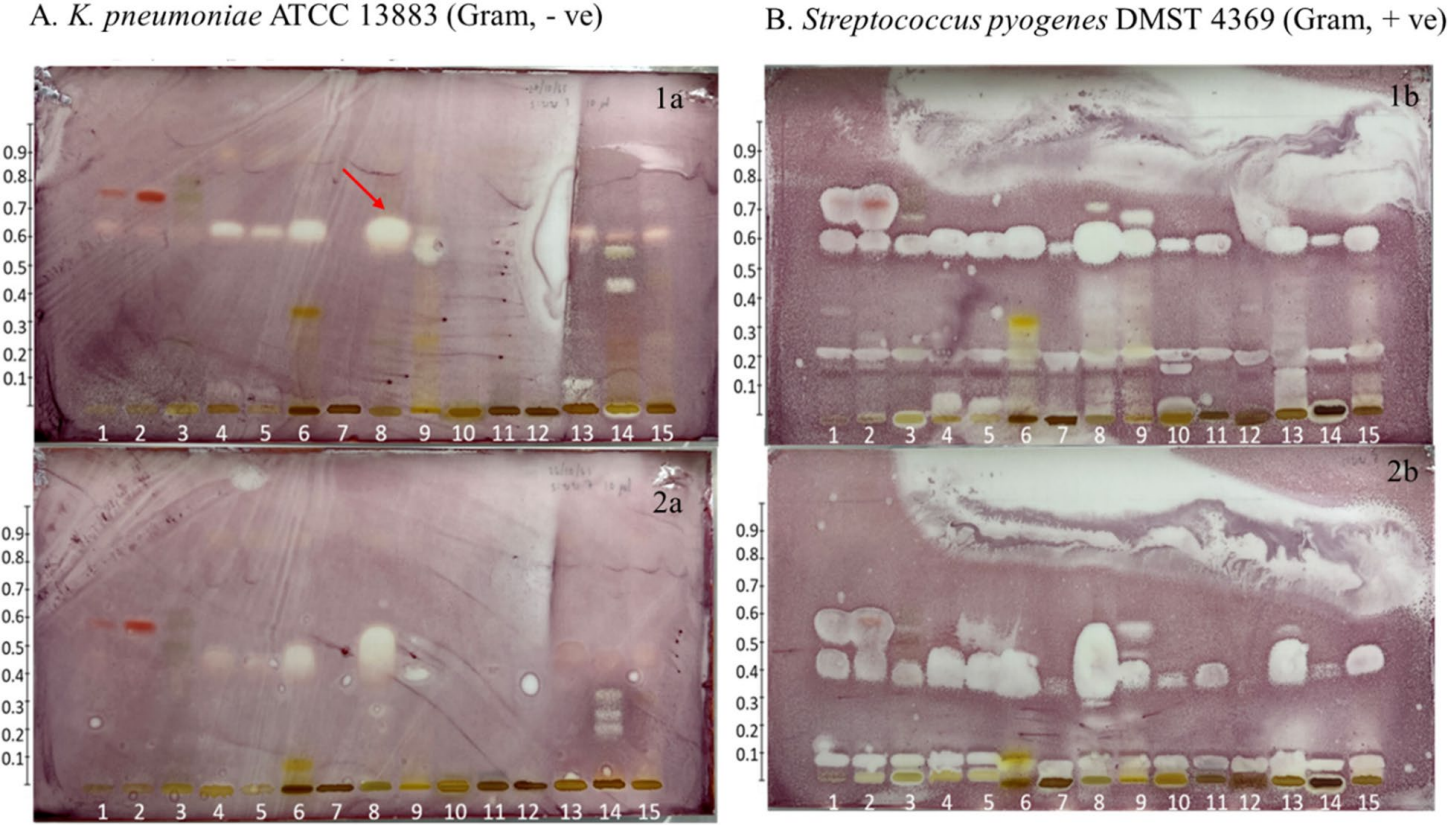
The inhibition zone of the YK formula and its crude drugs (1, the root of *S. androgynus*; 2, the root of *R. nasutus*; 3, the root of *M. vitifolia*; 4, the root of *D. volubilis*; 5, the root of *A. viridiflora*; 6, the root of *T. triandra*;* 7*, the root of *C. sinensis*; 8, YK; 9, the root of *C. aurantifolia*; 10, the root of *C. quadrangulare*; 11, the root of *M. cochinchinensis*; 12, the root of *G. zeylanicum*; 13, the root of *S. dichotomus/S. benthamianus*; 14, the stem of *H. formicarum*; 15, the root of *C. bonduc*) against **A** *K. pneumoniae* and **B** *S. pyogenes* on the TLC plate with different mobile phase systems: (1a and 1b) toluene, ethyl acetate, and formic acid (70:30:1) and (2a and 2b) hexane and ethyl acetate (2:1). The TLC plate incubated at 37 °C for 24 h, sprayed with 0.2% v/v INT dye to detect bacteria growth, and incubated for 1 h

TLC bioautography is a combination of TLC and antimicrobial activity assays to identify specific compounds with antibacterial activities. The results of the HPTLC mobile phases (toluene, ethyl acetate, and formic acid (70:30:1) and hexane and ethyl acetate (2:1)) were obtained by TLC bioautography. Two bacteria strains, *S. pyogenes* (Gram-positive) and *K. pneumoniae* (Gram-negative), were used. The inhibition zones of the YK formula and its crude drugs were found on both bacteria strains on TLC plates with different mobile phases (Fig. 4).

Regarding the inhibition zone on *K. pneumoniae*, the solvent developing system for the detection of apolar flavonoids (toluene, ethyl acetate, and formic acid (70:30:1)) identified an inhibition zone at R_f_ 0.61 in the methanolic extract of the YK formula, *D. volubilis*, *A. viridiflora*, and *T. triandra*, whereas inhibition zones were identified at R_f_ 0.55, 0.10, and 0.43 in *C. aurantifolia*, *S. dichotomus/S. benthamianus,* and *H. formicarum*, respectively (Fig. 4, 1a). Other solvent developing systems for the detection of naphthoquinones, such as hexane and ethyl acetate (2:1), identified an inhibition zone at R_f_ 0.40–0.60 in the methanolic extract of the YK formula, which is related to *T. triandra* and *C. aurantifolia* (R_f_ 0.45 and 0.40, respectively), whereas *H. formicarum* showed another three inhibition zones at R_f_ 0.20, 0.25, and 0.32 (Fig. 4, 2a).

Regarding the inhibition zone on *S. pyogenes*, an apolar flavonoid compound separated using the TLC mobile phase toluene, ethyl acetate, and formic acid (70:30:1) showed inhibition zones at R_f_ 0.20 and 0.55–0.62 in the methanolic extract of the YK formula and the methanolic extract of the crude drugs. Additionally, inhibition zones were identified at R_f_ 0.65–0.75 in *S. androgynus, B. androgyna,* and *R. nasutus* (Fig. 4, 1b). The naphthoquinone compound separated using the TLC mobile phase hexane and ethyl acetate (2:1) showed inhibition zones at R_f_ 0.08 and 0.30–0.56 in the methanolic extract of the YK formula, which is related to all crude drugs (Fig. 4, 2b).

### Effects of the YK formula extracts on the viability of RAW246.7 cells

**Fig. 5:**
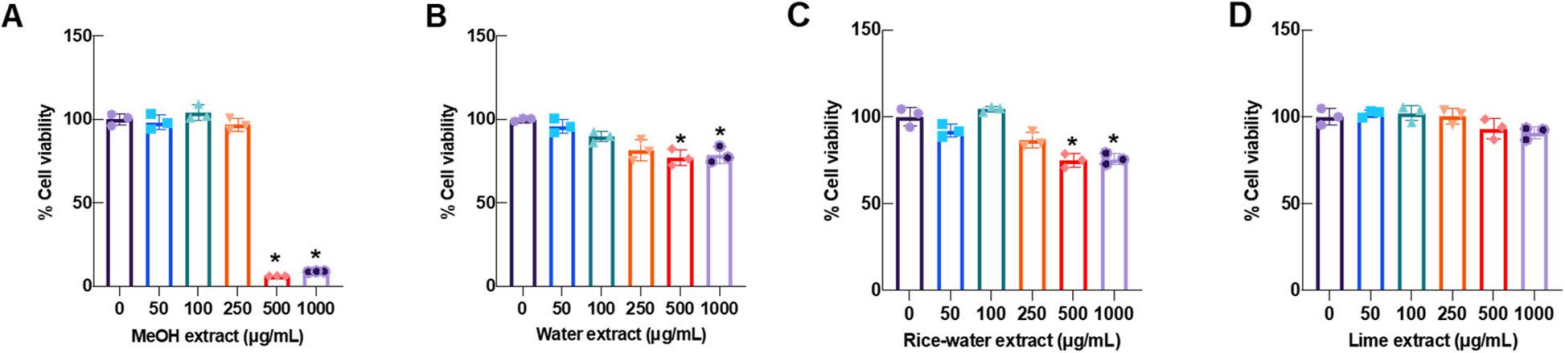
Evaluation of safety concentration of the YK formula extracts on RAW246.7 cells: MYK (**A**), WYK (**B**), RYK (**C**), and LYK (**D**) for 24 h. Following treatments, treated cells were determined using the MTT assay (*n* = 3; mean ± SD; ^*^p < 0.05 *vs.* untreated control)

Figure 5 shows the safety assessment of the YK formula extracts using four different solvents on RAW246.7 cells. The MTT results showed that the toxic effects of MYK, WYK, and RYK were observed at 500 µg/mL, whereas those of LYK were observed at 1,000 µg/mL. Only the CC50 (cytotoxic concentration 50%) value of MYK can be calculated to be 275.77 ± 17.92 µg/mL. Consequently, the safety concentration of the extract (≤ 250 µg/mL) was chosen for further evaluation of its anti-inflammatory properties.

### Effects of the YK formula extract on NO production in lipopolysaccharide (LPS)-induced RAW246.7 cells

**Fig. 6:**
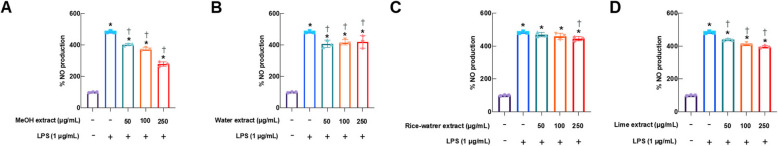
NO production in RAW264.7 cell supernatants after treatment with the YK extracts: MYK (**A**), WYK (**B**), RYK (**C**), and LYK (**D**) for 24 h. The treated cells were determined using Griess reagent (*n* = 3; mean ± SD; ^*^p < 0.05 *vs.* untreated control; ^†^*p* < 0.05 *vs.* LPS-treated cells)

NO is a reactive nitrogen species that plays a crucial role in the inflammatory response. Compounds capable of inhibiting NO production hold promise for potential anti-inflammatory applications. Prior to conducting the experiment, the experimental design was validated using hydrocortisone (100 µM) as a standard steroid control. Under the experimental conditions, NO production was significantly inhibited by approximately 10% by hydrocortisone (*p* < 0.01) (Supplementary Figure S4), supporting the validity of the experimental approach. The results showed that MYK, WYK, and LYK at concentrations ranging from 50 to 250 µg/mL effectively inhibited NO production in LPS-induced RAW246.7 cells. Regarding RYK, a significant reduction in NO production at *p* < 0.05 was observed only at a concentration of 250 µg/mL (Fig. 6). It is important to note that when the YK formula is traditionally administered, it is taken as a suspension where all components are consumed together. In this study, MYK was used as a representative to mimic the overall ingestion of the formula. The results indicate that MYK exhibited the strongest inhibitory effect on NO production compared to extracts prepared with other solvents, such as WYK, RYK, and LYK, which showed less pronounced effects. These results suggested that using water, rice-washing water, or lime juice to suspend YK in the traditional way did not significantly enhance its anti-inflammatory effects, compared to MYK, which demonstrated a clearly superior inhibition of NO production.

**Fig. 7:**
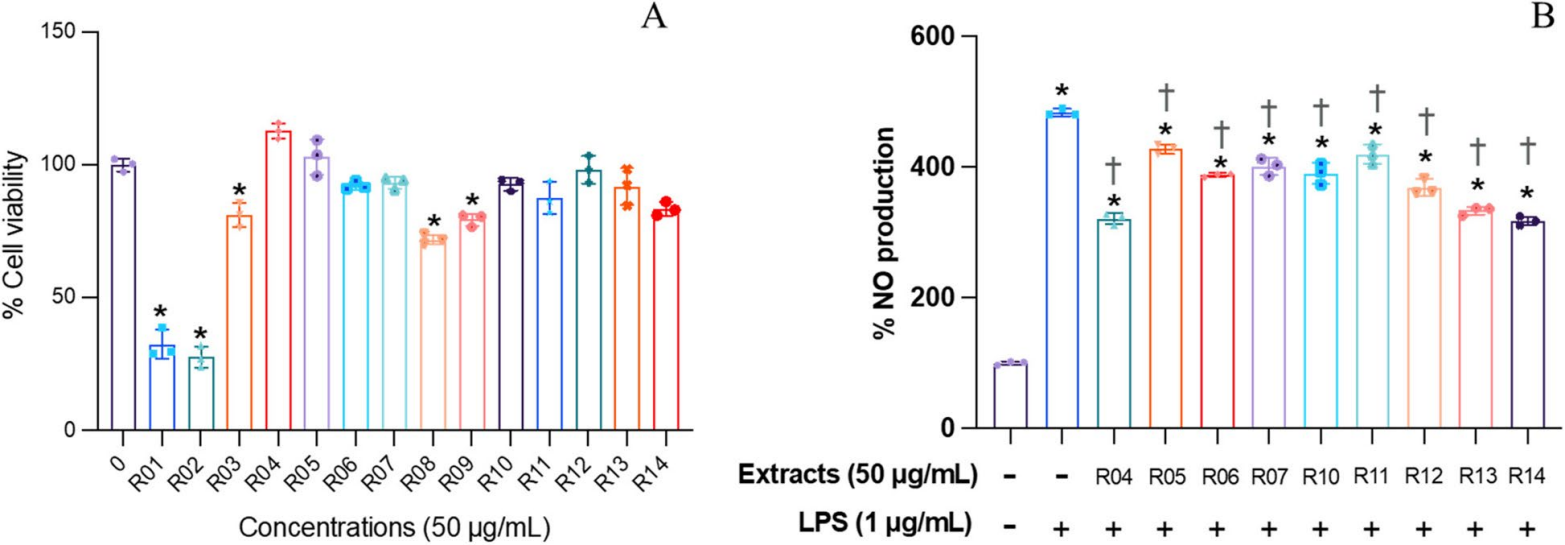
Effects of crude drug extracts on the viability of RAW264.7 cells (**A**) and NO production following LPS induction (**B**). The methanolic extract of R01, *S. androgynus*; R02, *R. nasutus*; R03, *M. vitifolia*; R04, *D. volubilis*; R05, *A. viridiflora*; R06, *T. triandra*; R07, *C. sinensis*; R08, *C. aurantifolia*; R09, *C. quadrangulare*; R10, *M. cochinchinensis*; R11, *G. zeylanicum*; R12, *S. dichotomus*; R13, *H. formicarum*; R14, *C. bonduc*. Cells were treated for 24 h, and the concentration of the individual extract was 50 µg/mL (*n* = 3; mean ± SD; ^*^p < 0.05 *vs.* untreated control; ^†^*p* < 0.05 *vs.* LPS-treated cells)

Furthermore, we conducted experiments to identify the specific crude drugs in the YK formula that contributed to the observed anti-inflammatory effects. The anti-inflammatory effects of the YK formula could be attributed to *D. volubilis*, *A. viridiflora*, *T. triandra*, *C. sinensis*, *M. cochinchinensis, G. zeylanicum, S. dichotomus/S. benthamianus, H. formicarum,* and *C. bonduc*. This result was supported by the significant inhibition of NO production (*p* < 0.05) in RAW246.7 cells induced by LPS observed with individual extracts from these crude drugs (Fig. 7B). Notably, extracts from *S. androgynus, B. androgyna, R. nasutus, M. vitifolia*, *C. aurantifolia,* and *C. quadrangulare* exhibited cytotoxicity, leading to their exclusion from anti-inflammatory assays (Fig. 7A).

## Discussion

The authenticity of medicinal plants is related to the effectiveness of the product. Therefore, it is important for herbal production. Several methods have been reported to identify medicinal plant authentication depending on the information needs. Macroscopic evaluation based on plant phenotypes is a method for identifying medicinal plant authentication using a professional taxonomist’s skills. However, a unique pattern of the characteristic compounds of a specific plant and the complex of herbal medicines could be observed by chemical fingerprinting [30]. In this study, combined methods were selected to authenticate medicinal plants in the YK formula. The first method used was a macroscopic evaluation by folk healers and experts from DTAM. The second method was an analysis of chemical fingerprinting by developing an HPTLC method. Some Thai plant and herbal medicine preparations have been reported in the THP.

In Thai history, medicinal plants used to treat plague were recorded in the Tak-Ka-Si-La textbook during the reign of King Chulalongkorn (the fifth monarch of Siam). The Tak-Ka-Si-La textbook describes fever symptoms related to those of COVID-19. Therefore, Thai folk medical wisdom suggests that YK relieves fever in patients with COVID-19 [31]. The YK formula contains 14crude drugs from 14 plants, and the identification method of some plants has been reported. Two mobile phases for HPTLC were developed to verify the authenticity of the crude drugs in the YK formula. The secondary metabolites in the crude drugs of the YK formula were detected by HPTLC and were assumed to be flavonoid and lignan compounds, as the mobile phase system and derivatizing agent used in the analysis were specific for these groups of compounds, although the exact identity of the compounds remains unknown. Previous studies have identified flavonoid compounds in *R. nasutus*, *C. aurantifolia*, *H. formicarum*, *S. dichotomus/S. benthamianus*, *C. sinensis*, *D. volubilis*, *G. zeylanicum*, *A. viridiflora*, *S. androgynus, B. androgyna*, *C. quadrangulare*, *M. cochinchinensis*, *T. triandra*, *C. bonduc*, and *M. vitifolia* [32–46], whereas lignans have accumulated in several plants in families such as Combretaceae, Cucurbitaceae, Rubiaceae, Apocynaceae [47, 48], and Acanthaceae (*R. nasutus*) [49]. For a better understanding of the active compounds in the YK formula, future studies should aim to identify the active compounds using other chromatographic techniques, such as High-Performance Liquid Chromatography (HPLC), Liquid Chromatography-Mass Spectrometry (LC–MS).

The YK formula is a mixture of dried crude drugs’ powder. The usage dose is 2 g of dried powder suspended in water. The powder should be suspended in rice water if the patient has a stuffy nose and mucus and in lime juice if the patient has a cough and sputum. The frequency of taking is 5 times per day every 3 h (06.00 A.M., 09.00 A.M., 12.00 P.M., 03.00 P.M., and 06.00 P.M.) for 5 consecutive days [4].

Fourteen plants in the YK formula do not have a report of their pharmacological activity related to COVID-19 infection. COVID-19 can lead to secondary infection through pathogenic bacterial infection and inflammation. Therefore, we investigated the antibacterial and anti-inflammatory activities of a combination of herbal drugs used for COVID-19 treatment to understand the pharmacological activity of the YK formula.

This study showed the antibacterial activity of MYK against Gram-positive bacteria, specifically *S. aureus* and *S. pyogenes*, which are known pathogens in upper respiratory tract infections [50, 51]. The observed antibacterial effect may be attributable to a synergistic combination of the plant constituents, including *C. quadrangulare*, *M. cochinchinensis*, *G. zeylanicum*, *H. formicarum*, *R. nasutus*, *S. androgynus*, and *M. vitifolia*. Additionally, LYK exhibited antimicrobial activity against Gram-negative bacteria, such as *K. pneumoniae* and *P. aeruginosa*, which are also implicated in upper respiratory tract infections [50]. In this study, antimicrobial activity was evaluated using the agar disc diffusion method. However, the minimum inhibitory concentration (MIC) and minimum bactericidal concentration (MBC) were not determined. Although the disc diffusion method is widely accepted for preliminary screening of antimicrobial activity, it does not provide quantitative data on the lowest concentrations required to inhibit or kill bacteria. MIC and MBC are critical for understanding the potency and efficacy of antimicrobial agents. Therefore, the absence of MIC and MBC data represents a limitation of this study. Future studies should determine the MIC and MBC of the YK extract and its individual plant components to provide a more comprehensive and quantitative assessment of their antimicrobial properties. This will help us deeply understand the extract’s therapeutic potential and make more precise comparisons with existing antimicrobial agents.

In this study, the TLC bioautography results showed antibacterial activities against *S. pyogenes* and *K. pneumoniae,* and the estimated compound groups were polar flavonoids, naphthoquinones, and lignans. Previous studies have shown the activity of the ethanolic *C. quadrangulare* root extract against several Gram-negative bacteria [52]. The activity of *C. quadrangulare* ripe fruit crude extract from methanol, hexane, acetone, and crude oil against *K. pneumoniae* and *P. aeruginosa* ATCC 27853 has been reported [53]. Additionally, the activity of the methanolic extract of *H. formicarum* tubers against *S. pyogenes* II with MIC at 256 ug/mL was previously reported [54]. The crude extract of *R. nasutus* root from hexane, methanol, ethanol, and chloroform has been reported to inhibit the growth of *S. aureus* ATCC 29213 (MIC 156.25–5,000 ug/mL) and β-*Hemolytic streptococci* (MIC 39.06–2,500 ug/mL) [55]. Particularly, 25 ug/mL rhinacanthin A and 25 ug/mL 3,4-dihydro-3,3-dimethyl-2*H*-naphtho[2,3-*b*]pyran-5,10-dione isolated from *R. nasutus* have been reported to inhibit the growth of *S. aureus* with inhibition zones of 16 and 20 mm [56]. These findings suggest that the combined effects of all the ingredients in the YK formula contribute to the inhibition of both Gram-positive and Gram-negative bacteria, which are common pathogens responsible for respiratory tract infections. However, further research is required to identify the specific active compounds responsible for these effects.

This study showed that MYK, WYK, and LYK had anti-inflammatory effects caused by nine root extracts, including *D. volubilis*, *A. viridiflora*, *T. triandra*, *C. sinensis*, *M. cochinchinensis, G. zeylanicum, S. dichotomus/S. benthamianus, H. formicarum*, and *C. bonduc*. Previous studies have shown that *D. volubilis, T. triandra*, *C. sinensis*, *M. cochinchinensis*, and *C. quadrangulare*, the main ingredients in Thai herbal drugs, namely Kerra capsules (the registration number G 40/57) used for relief fever, have anti-inflammatory effects [57]. Furthermore, mocochinoside A, triterpene glycosides such as momordin Ib, and calendulaglycoside C 6'-*O*-7-butyl ester isolated from *M. cochinchinensis* vine showed NO inhibition at IC_50_ as 5.41–11.28 µM [58]. 50 µg/mL of *S. androgynus, B. androgyna, R. nasutus, M. vitifolia*, *C. aurantifolia,* and *C. quadrangulare* extracts showed toxicity effects on RAW cells. However, low concentrations of these plants may exert anti-inflammatory effects. For example, the methanolic extract from *S. androgynus* leaves exhibited NO inhibitory activity at IC_50_ as 58.34 ± 1.11 µg/mL [59]. *R. nasutus* root and *C. aurantifolia* contain triterpenoids, which play a role as anti-inflammatory agents [60] through downregulation in the NF-kB signaling pathway [61]. Furthermore, essential oils (geranial compounds, limonene, and α-terpinene) from the peels of *C. aurantifolia* and the ethanolic extract of *C. aurantifolia* bark exert anti-inflammatory effects [33]. Leaf and stem extracts from *C. quadrangulare* inhibit the production of proinflammatory mediators through the mitogen‑activated protein kinase pathway [62]. Moreover, based on current findings, plant materials exhibiting both antibacterial and anti-inflammatory activities—such as *M. cochinchinensis*, *G. zeylanicum*, and *H. formicarum*—are likely to be key active components in the YK formula. These plants could potentially serve as markers for the standardization of the YK formula in the future.

Furthermore, some plants in the YK formula exhibit antiviral activity. β-Sitosterol from *S. androgynus* leaves demonstrated anti-dengue viral activities by inhibiting the fusion process during viral entry [13]. Rhinacasutone, rhinacanthone, rhinacanthins (C, D, N, Q, and E), heliobuphthalmin, and naphthoquinone racemate isolated from *R. nasutus* are effective against influenza PR8, HRV1B, and CVB3 virus [63]. Carboxylic acids, rhinacanthinic acids A–C, naphthoquinones, and lactones isolated from *R. nasutus* are effective against herpes simplex virus type 2 [64]. Furthermore, the ethanolic extract of *T. triandra,* the main ingredient in Ya-Ha-Rak, exerted effects against dengue virus serotype 2 [65]. Catechins, a major constituent of *C. sinensis*, inhibited SARS-CoV-2 through the model 3-chymotrypsin-like cysteine protease [66]. The hydroalcoholic extract of *C. sinensis* exhibits antiadenovirus activity via replication in the postadsorption stage [67]. Additionally, *C. quadrangulare* was reported to have antiviral activity [68]. Water-soluble gluco-arabinan from *C. bonduc* seeds, an alkaline extract, showed splenocyte and thymocyte stimulation, and arabinan showed appreciable macrophage activation [69]. In addition, there have been clinical studies on the use of YK for the treatment of post-COVID- 19 patients. Ya-Kao, also known as Ya-Krob-Khai-Taksila, is a traditional formula documented in Thai Traditional Medicine (TTM) textbooks. Ya-Kao is one of the top five most commonly used medicines in TTM clinics for the treatment of post-COVID- 19 conditions. The use of YK has been shown to significantly improve the quality of life of post-COVID- 19 patients [70].

Overall, the YK formula exhibited antibacterial and anti-inflammatory properties resulting from the synergistic effects of several plants in the formula. The results showed that MYK had the most effective antibacterial and anti-inflammatory activities compared with WYK, LYK, and RYK. Therefore, our findings suggested that YK should be taken following the traditional knowledge dosage form, which is a dry powder suspended in vehicle liquid solvents. The recommended liquid vehicle is lime juice because lime juice has better antibacterial activity. To the best of our knowledge, this is the first study to investigate the biological properties of the root and stem parts of each plant and the YK formula. Given the limitations of this study, continuous research on the YK formula is required to identify its major plant components and determine the key active compounds for use in the standardization process, which is essential for ensuring consistent quality and reproducibility. Moreover, additional clinical studies should be conducted in both healthy volunteers and patients with respiratory tract infections to comprehensively evaluate the formula’s safety, efficacy, and potential side effects. To ensure long-term patient safety, it is also important to implement pharmacovigilance strategies following clinical trials.

While the YK formula shows promising potential, challenges common to polyherbal formulations—such as variability in plant sources, possible herb-herb interactions, and regulatory difficulties—must be carefully considered. The data obtained from future studies would provide strong, evidence-based support for the clinical application of the YK formula. Such findings could be highly beneficial for practitioners of traditional Thai medicine as well as those in other alternative medical fields in the treatment of respiratory tract infections in the future.

## Conclusions

This study primarily focused on establishing quality control of the raw materials in the YK formula and revealing evidence supporting the extended benefit of YK in treating infection. We conducted an in vitro evaluation of its biological activity. The results showed that YK, a combination of 14 crude extracts, exhibited potential antibacterial and anti-inflammatory activities. *D. volubilis*, *A. viridiflora*, *T. triandra*, *C. sinensis*, *M. cochinchinensis, G. zeylanicum, S. dichotomus/S. benthamianus, H. formicarum,* and *C. bonduc* displayed NO reduction. YK demonstrated antibacterial effects against pathogenic bacteria in the respiratory tract. Particularly, *C. quadrangulare*, *M. cochinchinensis*, *G. zeylanicum*, *H. formicarum*, *R. nasutus*, *S. androgynus*, *M. Vitifolia*, and lime juice exhibited antibacterial activity against *S. aureus* and *S. pyogenes*. YK should be suspended in lime juice for a better antibacterial effect. Additionally, the anti-inflammatory effect of YK was observed through a reduction in NO synthesis in an in vitro macrophage model.

## Supplementary Information

Supplementary Material 1.

## Data Availability

Data are provided within the manuscript or supplementary information files.

## Abbreviations

YK: Ya-Kao
TLC: Thin-layer chromatography
HPTLC: High-performance thin-layer chromatography
NO: Nitric oxide
LPS: Lipopolysaccharide
TTM: Thai traditional medicine
WYK: Water extract of YK
LYK: Lime juice extract of YK
MYK: Methanol extract of YK
RYK: Rice-washing water extract of YK
MHA: Mueller–Hinton agar
MRSA: Methicillin-resistant *Staphylococcus aureus*
DMSO: Dimethyl sulfoxide
MHB: Mueller–Hinton broth
INT: Iodonitrotetrazolium chloride
ATCC: The American Type Culture Collection
MTT: 3-(4,5-Dimethylthiazol-2-yl)-2,5-diphenyltetrazolium bromide
NED: N- 1-naphthylethylenediamine dihydrochloride
THP: Thai Herbal Pharmacopoeia
