# Supplementary material for: Qualitative analysis and exploration of anti-inflammatory and antibacterial effects of a Thai traditional medicine formula from Wat Pho beyond its use for COVID-19 treatment
Source: BMC Complement Med Ther. 2025 Apr 25;25:159. doi: 10.1186/s12906-025-04873-3 (PMC12023551; doi:10.1186/s12906-025-04873-3)
Supplement: Supplementary file 1 — Supplementary Material 1. [file 12906_2025_4873_MOESM1_ESM.docx]

**Supplementary Figure**

**
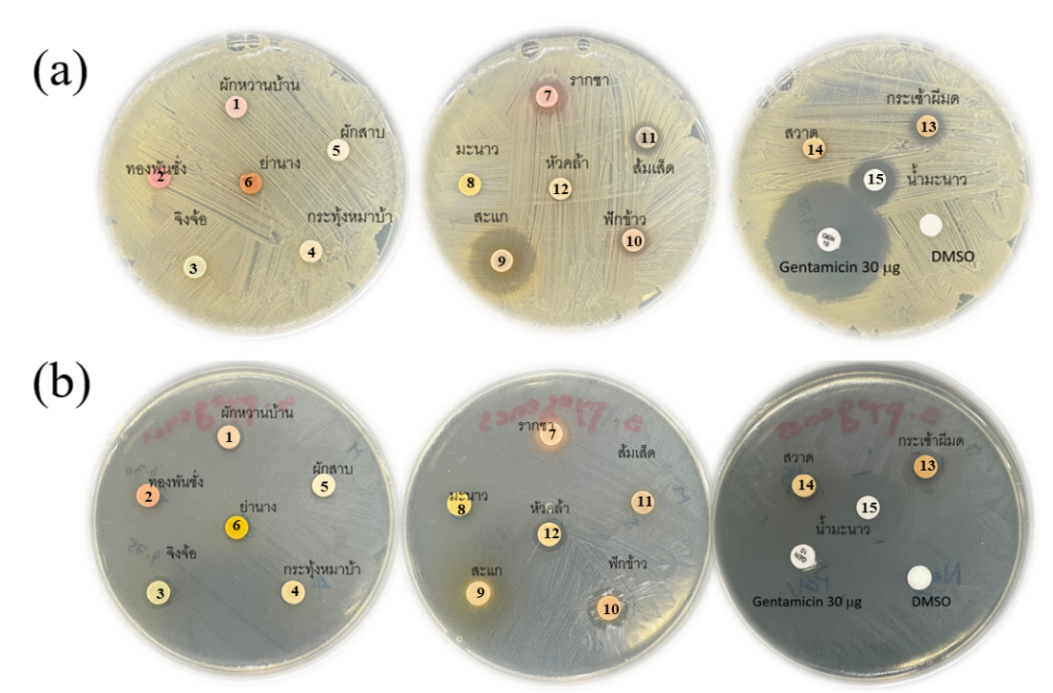
**

**Figure S1:** The inhibition zone of MYK extract, methanolic extract of the crude drug and lime juice at concentration of 100 mg/ml on *Streptococcus* species (a) *S. sorbinus* (b) *S. pyogenes* DMST 4369. The negative control and positive control were DMSO and Gentamicin, respectively.1, *S. androgynu;,*2, *R. nasutus;* 3, *M. vitifolia*; 4, *D. volubilis*; 5, *A. viridiflora*; 6, *T. triandra*; 7, *C. sinensis*; 8, *C. aurantifolia*; 9, *C. quadrangulare*; 10, *M. cochinchinensis*; 11, *G. zeylanicum*; 12, *S. dichotomus;* 13, *H. formicarum*; 14, *C. bonduc* and 15, lime juice.


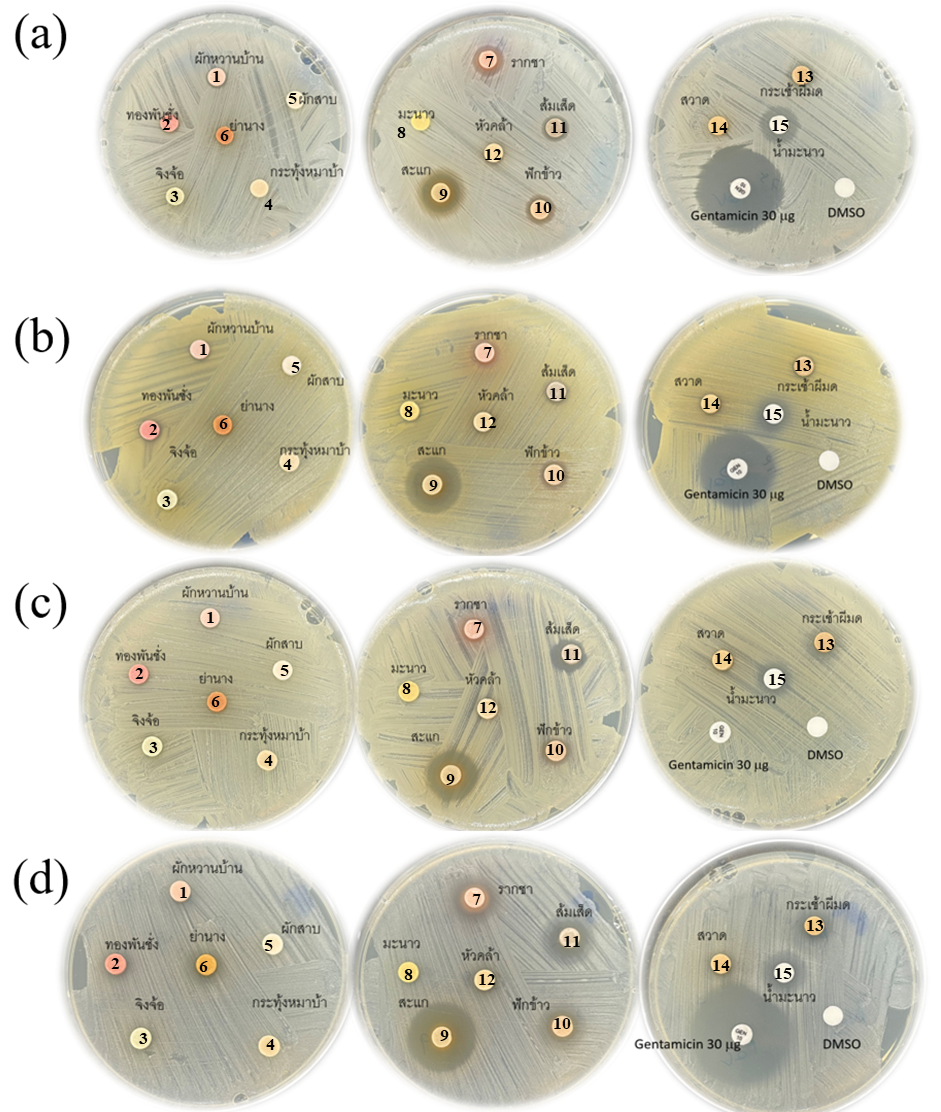


**Figure S2:** The inhibition zone of MYK extract, methanolic extract of the crude drug and lime juice at concentration of 100 mg/ml on *Staphylococcus species*; (a) *S. aureus* ATCC 25923; (b) *S. aureus* ATCC 6538; (c) methicillin*-*resistant *S. aureus* (MRSA) DMST 20646; and (d) *S. epidermidis* ATCC 12228. The negative control and positive control were DMSO and Gentamicin, respectively.1; *S. androgynus, B. androgyna,*2; *R. nasutus,* 3; *M. vitifolia*, 4; *D. volubilis*, 5; *A. viridiflora*, 6; *T. triandra*, 7; *C. sinensis*, 8; *C. aurantifolia*, 9; *C. quadrangulare*, 10; *M. cochinchinensis*, 11; *G. zeylanicum*, 12; *S. dichotomus/ S. benthamianus*, 13; *H. formicarum*, 14; *C. bonduc* and 15; lime juice.

**
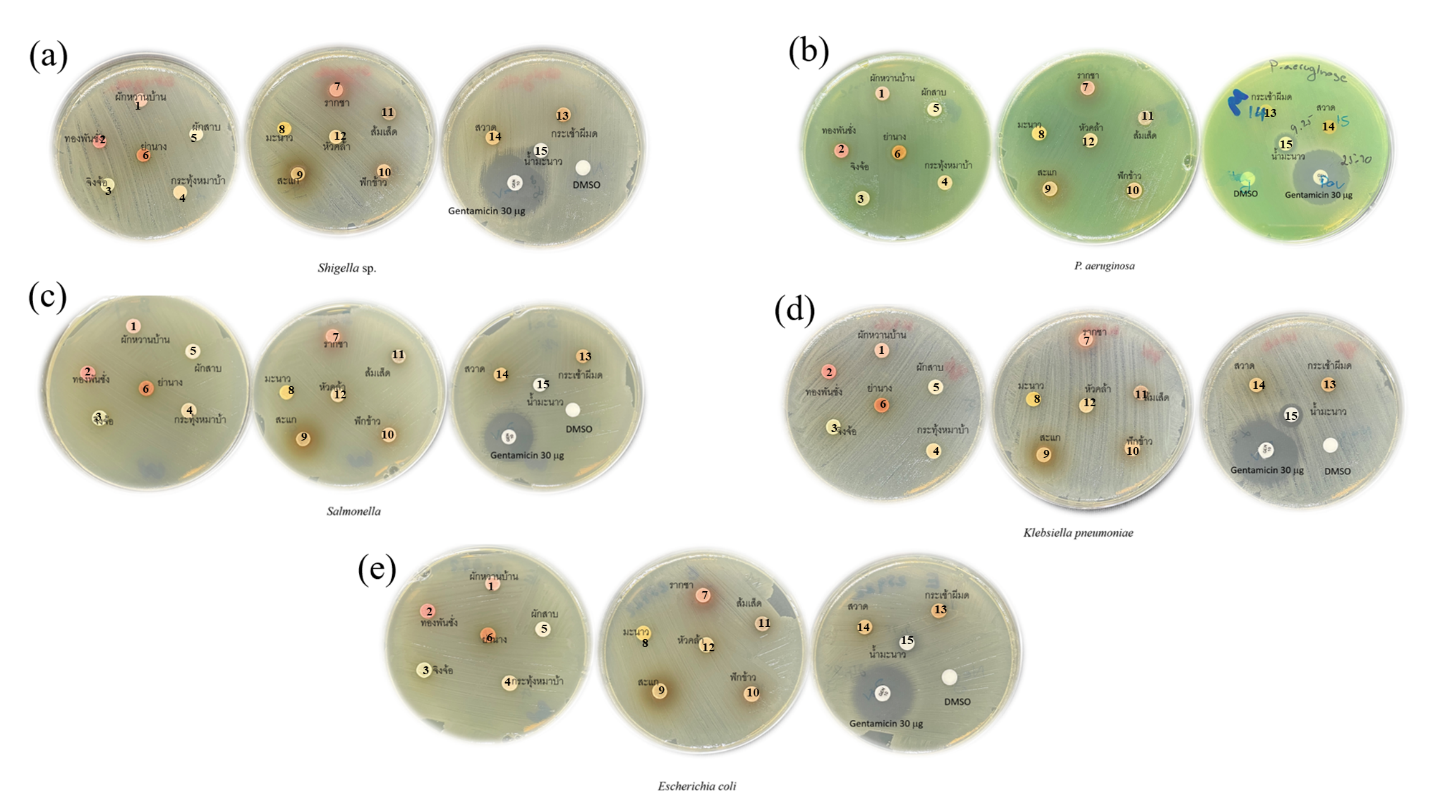
**

**Figure S3:** The inhibition zone of MYK formula, methanolic crude drug extract, and lime juice with concentration at 100 mg/ml on Gram negative bacteria strains; (a) *Shigella* sp. (b) *P. aeruginosa* (c) *Salmonella* sp. (d) *K.pneumoniae* and (e) *E.coli.* The negative control and positive control were DMSO and Gentamicin, respectively.1; *S. androgynus, B. androgyna,*2; *R. nasutus,* 3; *M. vitifolia*, 4; *D. volubilis*, 5; *A. viridiflora*, 6; *T. triandra*, 7; *C. sinensis*, 8; *C. aurantifolia*, 9; *C. quadrangulare*, 10; *M. cochinchinensis*, 11; *G. zeylanicum*, 12; *S. dichotomus/ S. benthamianus*, 13; *H. formicarum*, 14; *C. bonduc* and 15; lime juice.

**Figure S4:** Hydrocortisone suppress the NO production of LPS-induced-RAW cells. Raw cells were exposed to LPS in the presence/absence of hydrocortisone for 24 h. Following treatments, NO level was evaluated (n = 3; mean ± SEM ****, p < 0.0001 v.s. untreated control; †† p < 0.01 v.s. LPS-treated cells).
